# Donor Human Milk: Effects of Storage and Heat Treatment on Oxidative Stress Markers

**DOI:** 10.3389/fped.2018.00253

**Published:** 2018-10-05

**Authors:** Enrico Bertino, Chiara Peila, Francesco Cresi, Elena Maggiora, Stefano Sottemano, Diego Gazzolo, Sertac Arslanoglu, Alessandra Coscia

**Affiliations:** ^1^Neonatology Unit, Department of Public Health and Pediatrics, Università degli Studi di Torino, Turin, Italy; ^2^Department of Maternal, Fetal and Neonatal Health, Azienda Ospedaliera Nazionale SS. Antonio e Biagio e Cesare Arrigo, Alessandria, Italy; ^3^Neonatal Intensive Care Unit, Università degli Studi G. d'Annunzio Chieti e Pescara, Chieti, Italy; ^4^Department of Pediatrics, Division of Neonatology, Istanbul Medeniyet University Goztepe Education and Research Hospital, Istanbul, Turkey

**Keywords:** donor human milk, human milk, human milk bank, oxidative stress, heat treatment, refrigeration, pasteurization

## Abstract

Mother's own milk is the first choice for the feeding and nutrition of preterm and term newborns. When mother's own milk is unavailable or in short supply donor human milk (DM) could represent a solution. Heat treatment and cold storage are common practices in Human Milk Banks (HMBs). Currently, Holder pasteurization process is the recommended heat treatment in all international guidelines. This method is thought to lead to a good compromise between the microbiological safety and nutritional/biological quality of DM. Moreover, storage of refrigerated milk is a common practice in HMBs and in NICUs. Depending on the length and on the type of storage, human milk may lose some important nutritional and functional properties. The available data on oxidative stress markers confirm that pasteurization and refrigeration affected this important elements to variable degrees, even though it is rather difficult to quantify the level of deterioration. Nonetheless, clinical practice demonstrates that many beneficial properties of human milk are preserved, even after cold storage and heat treatment. Future studies should be focused on the evaluation of new pasteurization techniques, in order to achieve a better compromise between biological quality and safety of DM.

## Introduction

Human milk (HM) is the gold standard for feeding and nutrition for preterm and term newborns. Mother's own milk is the first choice for improving short and long-term outcomes for all neonates (1–3). HM benefits are mediated by different components, including specific and immunomodulatory molecules and species-specific factors. Breastmilk could be considered as a dynamic system and species-specific nourishment for newborns (4). Human Donor Milk (DM) can replace breastmilk when unavailable or lacking, although safe procedures for milk storage and conservation are required (5, 6). Currently, several reviews that show the advantages of donor milk, the World Health Organization and the American Academy of Pediatrics suggest the use of DM as a substitute of mother milk (1, 3).

Heat treatment on milk delivered to Human Milk Banks (HMBs) is critical for milk safety: pasteurization, indeed, inactivates bacterial and viral agents (5). Pasteurization is a process consisting of three main phases: rapid heating, stationary temperature phase and rapid cooling. Currently, the Holder Pasteurization (HoP) method, providing a temperature of 62,5°C for 30 min, is fundamental for HMBs constitution and its use is suggested by international guidelines (5, 6) as HoP represents the best compromise between nutritional and biological characteristics and microbiological safety (7–11).

Cold storage of HM is a routine not only in HMB but also at home and in the hospitals, especially in Neonatal Intensive Care Units. According to the length and on the typology of storage, HM may lose some significant nutritional and functional characteristics. The maximum refrigeration time for human milk ranges between 24 h and 8 days, according to the current advices on safe HM storage (12–14). Such variability replicates the heterogeneity of the scientific sources, which is ascribable to differences in the study design and in methodological approach (5, 14, 15). Recently, Slutzah et al. concluded that HM may be stored for 96 h at 4°C without affecting the overall milk integrity, as determined by bacterial growth, white cell count, pH, osmolality, and concentration of selected biological factors (sIgA, lactoferrin, total fat, and total proteins) (16, 17).

On the other hand, detailed data on the effects of storage, in terms of oxidative stress markers, on mother's milk are still lacking. Thus, the present paper is aimed at reviewing published evidences, and at comparing results on the effects of HoP and the refrigerated storage on the oxidative stress markers of human milk.

## Search methodology

The literature review was performed by electronic searches of MEDLINE, EMBASE, CINHAL, and the Cochrane Library. The electronic search used the following keywords and MeSH terms: donor milk, banked milk, milk bank, milk banking, (human milk OR donor milk) AND Holder pasteurization AND oxidative stress, (human milk OR donor milk) AND pasteurization AND oxidative stress, (human milk OR donor milk) AND storage AND oxidative stress, (human milk OR donor milk) AND heat treatment AND oxidative stress, (human milk OR donor milk) AND cold treatment AND oxidative stress, (human milk OR donor milk) AND refrigeration AND oxidative stress(donor milk OR Holder pasteurization) AND oxidative stress, (donor milk OR cold storage) AND oxidative stress, (donor milk OR cold storage) AND oxidative stress.

The research was performed in December 2017 and no limits concerning publication date were set.

Considering differences between the research protocols published to date, we focused our review on studies with an experimental design that:

- define exactly the pasteurization method (62.5–63°C for 30 min)
- define exactly the refrigeration method (4°C for maximum 96 h)
- compare the same samples of HM before and after the heat or the cold treatments.

## Heat treatment

The effects of HoP on oxidative stress markers are evaluated only in three studies.

Oxidative status was assessed, on raw and pasteurized breastmilk, by the evaluation of oxidants molecules and the activity of oxidants scavengers. Silvestre et al. showed that HoP does not significantly influence the levels of malondialdehyde while glutathione peroxidase activity, glutathione concentrations and total antioxidant capacity result seriously compromised. This result shows a reduction on oxidative scavenging systems of HM (18). Other authors (19) did not find changes in hexanal and malondialdehyde concentration and in the total antioxidant capacity (measured by means of oxygen radical absorbance capacity essay) thus meaning no lipid oxidation neither contraction of antioxidant systems.

Moreover, Peila et al. analyzed the effects of HoP on an emergent oxidative stress marker of the HM: the hemeoxygenase-1 (HO-1) (20). HO-1 is a stress-inducible rate-limiting enzyme and it is involved in different cytoprotective effects, due to its multiple catalytic by-products. HO-1 is active in HM and shows no significant reduction in its activity after HoP process, even after being corrected for milk maturation degree and gestational age (20). The protective effect of HO-1, similarly to other milk antioxidant scavenger systems, could be found in its antioxidant activity which induces the conversion of free heme into three final products: (i) biliverdin, which is metabolized in bilirubin that shows antioxidant activity), (ii) carbon monoxide, a neurotransmitter and vasodilator with antiapoptotic and anti-inflammatory activities, and (iii) iron (Fe^2+^) which is bound by specific proteins (20–24). Furthermore, HO-1 could have an immunoregulatory role in addition to its enzymatic activity, related to its capacity to bind specific receptors and to modulate the immune response (20). Indeed, extracellular stress proteins, including Heat Shock Proteins (HSP), rise as fundamental mediators of signaling and transport (25, 26). Behavioral stress influences the release of this proteins by the cell as well as the exposition to immunological “danger signals” (24). HSP released into extracellular fluid can bind receptors exposed by adjacent cells and begin the signal transduction cascades, likewise, the transport of molecules like antigenic peptides and chaperokines with immunomodulatory effect (20, 27). In particular, Li Volti et al. through a molecular modeling approach, found an important immunoregulatory receptor that could be the natural ligands of HO-1(20, 28). Nevertheless, the integration of experimental data with informatics data shows HO-1 role in the modulation of immune system (28).

Considering the various functions of HO-1 in the body, data reported in literature showing underlying a reduction in NEC incidence in preterm fed with DM compared to those fed with formula (7–11) and, the unclear pathophysiology of NEC (immature gastrointestinal epithelium, impaired immunological defenses, enteral feeding, and bacterial colonization), it is possible to argue that human milk HO-1 may play a role in the development and regulation of the immune system of the gastrointestinal tract (20).

Over the past decades the food industry and, in particular, the dairy industry tested innovative alternatives to standard pasteurization in an effort to maximize the preservation of food taste and nutritional features. Alternative processing techniques that are currently being tested to investigate their effect on HM include high-temperature–short-time pasteurization (HTST), high pressure processing (HPP), ultraviolet (UV) irradiation and (thermo-) ultrasonic processing (29).

HTST is a thermal pasteurization method that is well established in the dairy industry (“fresh” bovine milk is usually pasteurized by means of HTST). The method involves a thin layered milk flow being heated rapidly to 72°C and being kept at this temperature for a few seconds (usually 15 s), and then immediately cooled down. This method preserves most of the sensory features and nutritional values of the milk, and ensures a lower degradation of proteins and vitamins (29). Silvestre et al. (18) investigated oxidative stress markers (reduced glutathione, glutathione peroxidase activity, malondialdehyde, and total antioxidant capacity), and showed that the pasteurization of HM implies a decrease in its antioxidant properties, especially in the glutathione balance, but HTST caused a smaller loss in antioxidant potential than HoP.

## Cold storage

The effects of refrigerated storage at 4°C on oxidative stress markers are evaluated only in four studies.

Concerning the insurgence of lipid peroxidation and the creation of oxidation molecules, contrasting data have been reported in literature (30–32). Some studies reveal that cold storage of HM may reduce its antioxidants capacity (32) and increase malondialdehyde concentration (30). On the other hand, the study of Giribaldi et al. showed that the refrigerated storage at 4°C for 96 h did not affect the oxidative status of HM, evaluating the total antioxidant capacity, conjugated dienes, thiobarbituric acid reactive species and malondialdehyde concentration (14, 17). Their results did not indicate any evidence of lipid peroxidation, same Michalski et al reported (17, 31). The oxidative status of the HM during cold storage is particularly relevant for preterm newborns whose disorders are mainly due to disequilibrium between antioxidant capacity and oxidative stress, having a reduced antioxidant capacity and being often exposed to oxidant stress (32, 33). Moreover, the recent study of Peila et al. evaluated the effects of prolonged refrigerated storage on an important marker in HM: Adrenomedullin (AM) (34). AM is a C-amidated peptide, implicated in response to hypoxia and inflammation, which are linked also with neovascularization. Recent studies indicate that AM is synthesized also in the mammary gland and secreted in breast milk (35–37). AM levels in preterm milk (milk produced to mother who have delivered preterm, Gestational Age <37 weeks) are significant higher compared to term milk (milk produced to mother who have delivered at term, Gestational Age >37 weeks). This protein is not thermostable at 4°C: AM is significant reduced (56%) at 24 h and is nearly undetectable at 96 h (34). AM is a regulatory peptide and its expression was demonstrated in several tissues and biologic fluids such as plasma, cerebro-spinal fluid, sweat, amniotic fluid and urine (38–40). AM has been involved in the modulation of several physiological functions including cardiovascular tone, central brain activity (41–44), bronchodilation, renal function, hormone secretion, cell growth-differentiation, and immune response(45–52). Moreover, AM has been tested for its connection to ischemia-reperfusion injury whilst in healthy infants has been proved to contribute in the cascade of events sustaining fetal/neonatal cardiovascular adaptation (43–47). AM has been also taken into consideration for the analysis of beneficial/side-effects of *in-utero* vasodilation therapeutic strategies in pregnancies complicated by fetal chronic hypoxia (43–47). Concerning this, relation with AM and the occurrence of adverse neurological outcome has been reported in infants with congenital heart disease (47). Considering these important functions, it is possible to hypothesize that the existence of the active peptide AM in HM, and its variability in concentrations throughout different milk maturation degrees, gestational pathologies and gestational age at delivery, could have some direct impact in infants development due to the various physiological activities that have been related to it. In the gastrointestinal tract, immunoreactive AM has been found in human stomach, duodenum, jejunum, ileum and colon (47, 53, 54), and specific binding sites have been also found in rat stomach (54). This arrangement supposes a role for AM in the regulation of secretory-motor functions in the gastrointestinal tract, as well as in its development during the embryogenesis and the period immediately following birth. Since the developing intestine in the neonate is believed considered to be one of the main target organs for the growth factors present in human milk, Pio et al. proved that milk has a growth-promoting function on human small intestinal epithelial cell line (Int-407) (35, 36). These authors hypothesize that since MoAb-G6 partially blocks the milk-induced growth, AM may be one of the growth factors present in milk (35, 36). AM has also been characterized as an agent with antimicrobial activity against gastrointestinal microorganisms (36, 51, 52). This activity could be relevant for the protection of the neonate against gastroenteritis produced by intestinal pathogens. Eventually, since some peptides are absorbed from the neonatal gastrointestinal tract and appear intact in plasma (36, 55), AM could also exercise an activity in the modulation of tissue growth as well as in the regulation of the immune system (36, 55).

## Conclusion

Multiple studies have been conducted to evaluate the effects of pasteurization and cold storage on breast milk and the results indicate that these treatments affect the concentration and activity of the constituents of HM to varying degrees. However, many studies show the persistence of the benefits of donated milk compared to artificial milk in the nutrition of preterm infants. With regard to the effects on oxidative stress markers, the data are currently lacking and contrasting. Many questions remain to be answered in particular, future studies will have to be conducted to clarify the aspects not yet investigated on the markers of oxidative stress and on biological properties in relation to the HoP and cold treatments of breast milk. Future investigations must be aimed at improving the biological quality and safety of DM and should be: (i) designed to investigate the pre-analytical stability of these components according to storage procedures; (ii) intended to evaluate innovative test technologies, such as metabolomics; (iii) focused on new pasteurization techniques (high-temperature short-term pasteurization, thermoultrasonic treatment, high-pressure processing, and Ohmic heat treatment); (iv) aimed to evaluate analytical techniques able to assess the protein changes due to thermic treatments, as well as their interaction with sugars and lipids; (v) evaluated the effects of HoP on other biomarkers involved in growth and developing of newborns.

Moreover further studies will aimed at elucidating the protein stability during industrial processes for the preparation of artificial milk such as pasteurization and spray-drying, which have already been shown to affect milk composition and properties.

## Author contributions

EB, CP, and AC contributed conception and design of the review. CP wrote the first draft of the manuscript. CP, EM and SS wrote sections of the manuscript. All authors contributed to manuscript revision, read, and approved the submitted version.

### Conflict of interest statement

The authors declare that the research was conducted in the absence of any commercial or financial relationships that could be construed as a potential conflict of interest.
